# Preparation and evaluation of fenbendazole methyl-*β*-cyclodextrin inclusion complexes

**DOI:** 10.1186/s12917-024-04056-1

**Published:** 2024-05-20

**Authors:** Yili Ding, Zhiyuan Zhang, Charles Ding, Shufeng Xu, Zhe Xu

**Affiliations:** 1 College of Science, Mathematics and Technology, Wenzhou-Kean University, Wenzhou, 325060 P. R. China; 2 Dorothy and George Hennings College of Science, Mathematics and Technology, Kean University, 1000 Morris Ave, Union, NJ 07083 USA; 3 Life Science Department, Foshan University, Foshan, Guangdong P. R. China 528000; 4 Keck School of Medicine of USC, Los Angeles, CA 90089 USA

**Keywords:** Fenbendazole, Methyl-*β*-cyclodextrin, Inclusion complex, Water solubility, *In vitro* and *in vivo* pharmacokinetic study

## Abstract

As an orally effective benzimidazole anthelmintic agent, fenbendazole was not only widely used in agriculture and animal husbandry to prevent and treat parasites, but also shows anti-cancer effects against several types of cancer, exhibits anti-cancer effects in paclitaxel and doxorubicin-resistant cancer cells. However, fenbendazole’s poor in water solubility (0.3 µg/mL), limits its clinical applications. Even great efforts were made toward increasing its water solubility, the results were not significant to reach anti-cancer drug delivery requirement (5–10 mg/mL). Through single factor and orthogonal strategy, many complex conditions were designed and used to prepare the complexes, the inclusion complex with methyl-*β*-cyclodextrin with 29.2 % of inclusion rate and 89.5% of inclusion yield can increase drug’s water solubility to 20.21 mg/mL, which is the best result so far. Its structure was confirmed by differential scanning calorimetry, scanning electron microscopic image, 1D and 2D NMR spectra in D_2_O. In its *in vitro* pharmacokinetic study, fenbendazole was 75% released in 15 min., in its *in vivo* pharmacokinetic study, the bio-availabilities of fenbendazole, its major metabolic anthelmintic agent oxfendazole and its minor metabolic anthelmintic agent oxfendazole were increased to 138%, 149% and 169% respectively, which would allow for fewer drug doses to achieve the same therapeutic effect and suggest that the complex can be used as a potential anticancer agent.

## Introduction

Fenbendazole (1) as an orally effective benzimidazole anthelmintic agent with broad antiparasitic activity, high efficiency, and low toxicity was widely used in agriculture and animal husbandry to prevent and treat parasites [1]. More important, as a microtubule destabilizing agent, fenbendazole acts on worms primarily by binding to tubulin and disrupting tubulin-microtubule balance, stabilizes the transcriptional activator HIF-1α, causes cell cycle arrest and mitotic cell death, shows anti-cancer effects against several cancer types [2–4] and exhibits anti-cancer effects in paclitaxel and doxorubicin-resistant cancer cells [5, 6]. However, fenbendazole is insoluble in water (0.3 µg/mL) [7], belongs to the IV class of drugs with low permeability and solubility according to the biopharmaceutical classification of FDA [8]. An anti-cancer drug delivered by IP or IV route requires usually 5–10 mg/mL aqueous solubility, since low concentration of fenbendazole would require large volume to reach the required therapeutic dose. Low water solubility and low bioavailability limit fenbendazole’s clinical application and enhancing its water solubility can effectively improve its medical applications.

Cyclodextrins were used to form the inclusion complexes with fenbendazole to change its physicochemical properties and improve its wettability, dissolution and stability. Through aqueous solution method, fenbendazole was complexed with 2-hydroxypropyl-*β*-cyclodextrin or *β*-cyclodextrin, and the aqueous solubility of fenbendazole was found as 40 µg/mL in solution of *β*-cyclodextrin (4% w/v) or 100 µg/mL in solution of 2-hydroxypropyl-*β*-cyclodextrin (10% w/v) respectively [9], by using similar conditions, the complex with *β*-cyclodextrin was prepared and could increase the water solubility of fenbendazole to 45.56 µg/mL, while complexation with 2-hydroxypropyl-*β*-cyclodextrin could increase the water solubility of fenbendazole to 159.36 µg/mL [10].

Besides cyclodextrin complexations, many other strategies were used to increase fenbendazole’s water solubility. Mesylate and tosylate salts of fenbendazole were prepared for increasing fenbendazole’s water solubility [11], supramolecular complex of fenbendazole with polyvinylpyrrolidone polymer could increase fenbendazole's solubility by 2.8 times [12], self-dispersible nanocrystals of fenbendazole prepared from fenbendazole and Poloxamer 188 in 1:1 proportion was used for fenbendazole’s *in vivo* PK study [13]. Fenbendazole loaded with Mobil composition of matter number 41 showed enhanced delivery capability to PC-3 cells [14], the micelles containing fenbendazole and rapamycin (1:2 ratio) prepared via freeze-drying showed slower release *in vitro* [15], fenbendazole-encapsulated poly-(D,L-lactide-co-glycolide) acid nanoparticles prepared by an oil-in-water emulsion method [16] could increase water solubility, enhance absorption, and exert significant anti-cancer effects in EOC cells and xenograft models including PDX [17], fenbendazole dispersed in PEO/PCL blend-based matrices prepared by hot-melt extrusion revealed that PCL retarded the drug release proportionally to the content of such polymer incorporated [18]. Compatibility between fenbendazole and three polymeric excipients was evaluated [19], and tablets of plasticized solid dispersions of poly-(ethylene oxide)/polycaprolactone and fenbendazole improved the drug solubility and release [20]. These research works did not make significant solubility improvement and could not reach the requirements for a cancer therapeutic dose (5–10 mg/mL), therefore, more effects need to be put to find new formulation with significant water solubility increasing [21–24].

Through the complexation with cyclodextrins under many different conditions, the obtained inclusion complex could increase the water solubility of fenbendazole as high as 20.21 mg/mL. In this communication, we like to report these results in detail, and it’s *in vitro*, *in vivo* pharmacokinetic studies.

## Results and discussion

**Fig. 1:**
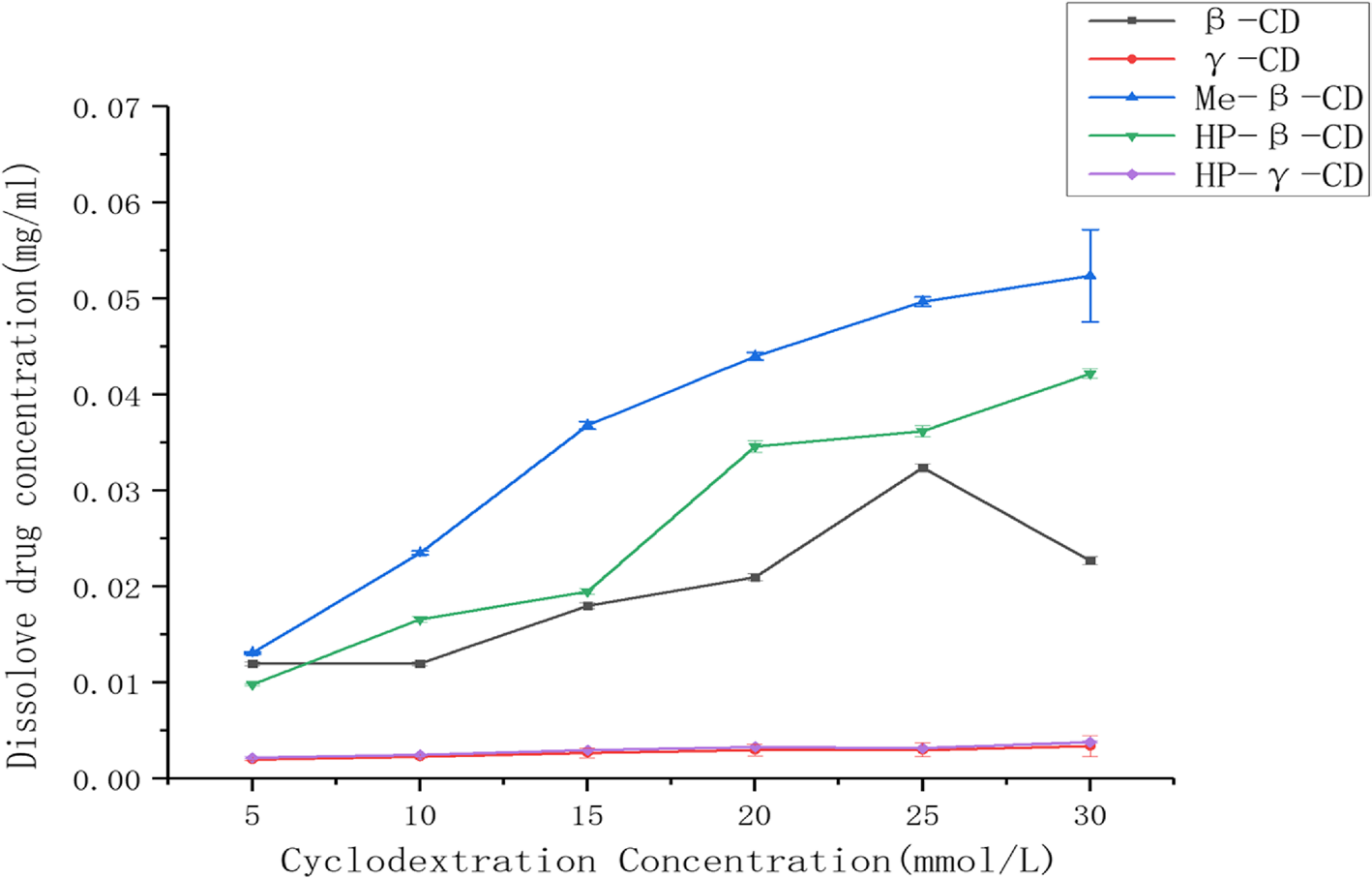
The solubility of fenbendazole in aqueous solutions of *β*-cyclodextrin, *γ*-cyclodextrin, 2-hydroxypropyl-*β*-cyclodextrin, 2-hydroxypropyl-*γ*-cyclodextrin and methyl-*β*-cyclodextrin with different concentrations

When extra fenbendazole was added to the aqueous solutions of *β*-cyclodextrin, 2-hydroxypropyl-*β*-cyclodextrin, methyl-*β*-cyclodextrin, *γ*-cyclodextrin or 2-hydroxypropyl-*γ*-cyclodextrin with concentrations of 5 mmol/L, 10 mmol/L, 15 mmol/L, 20 mmol/L, 25 mmol/L or 30 mmol/L respectively, the mixtures were shake at room temperature for 48 hours, filtered and analyzed by HPLC with UV detector at 296 nm wavelength, and the solubility for each mixture was obtained (Fig. 1) based on HPLC standard curve and regression equation, from which methyl-*β*-cyclodextrin was selected to evaluate the complexation conditions with fenbendazole further. Ratio of the fenbendazole and methyl-*β*-cyclodextrin, concentration of methyl-*β*-cyclodextrin, complexation temperature, stirring speed, reaction time and complexation method were considered to affect the results. Through single factor and orthogonal strategy, many reaction conditions were designed and used to prepare the complex. When the mixture of fenbendazole and methyl-*β*-cyclodextrin (1:1 ratio) in water was stirred at 50 °C for 3 hours with 500 r/min., the inclusion complex with 29.2 % of inclusion rate and 89.5% of inclusion yield was obtained with the highest water solubility of fenbendazole so far as 20.21 mg/mL, which is 60000 times better than that of fenbendazole alone. This complex was used to confirm the formation of the inclusion complex, and *in vitro* and *in vivo* PK study.

**Fig. 2:**
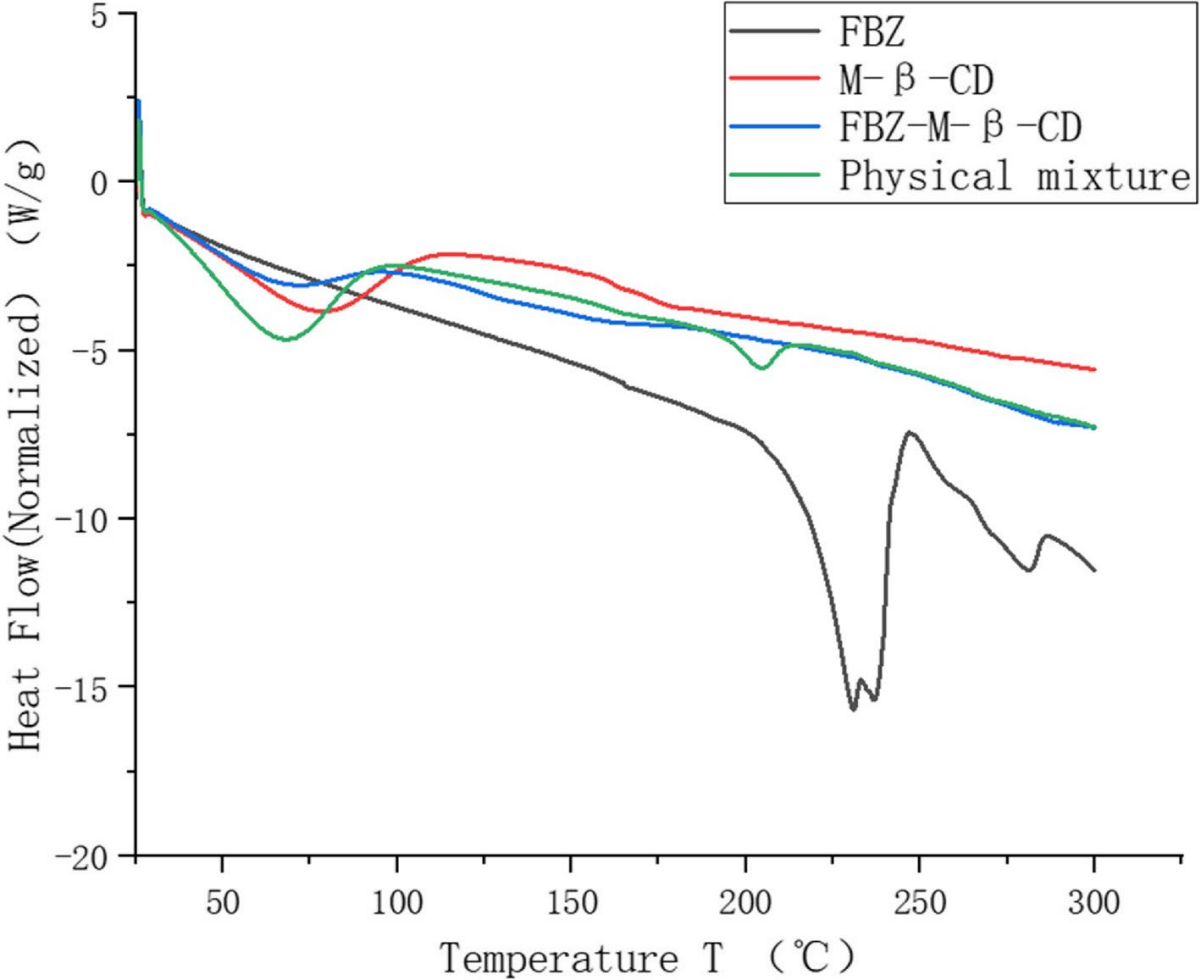
Differential scanning calorimetry of methyl-*β*-cyclodextrin, fenbendazole, their physical mixture and inclusion mixture

The differential scanning calorimetry (DSC) was used to confirm the formation of the inclusion complex (Fig. 2). The endothermic event in the curve of methyl-*β*-cyclodextrin in range 50–125℃ was due to the water loss from its cavity, in the curve of fenbendazole, the endothermic events at 227℃ and 237℃ were seen due to the melting effects of two tautomeric forms, in the curve of the physical mixture, the endothermic event in the range 50–125℃ was due to the dehydration of methyl-*β*-cyclodextrin, the absence of the endothermic events at 227℃ and 237℃ and the presence of an endothermic event at 200℃ indicated the interactions between fenbendazole and methyl-*β*-cyclodextrin in the physical mixture. In the curve of the inclusion complex, the same thermal profile as methyl-*β*-cyclodextrin was presented probably due to the extra methyl-*β*-cyclodextrin in the complex, the peaks of fenbendazole at 227℃ and 237℃ were disappeared due to the formation of the inclusion complex.

**Fig. 3:**
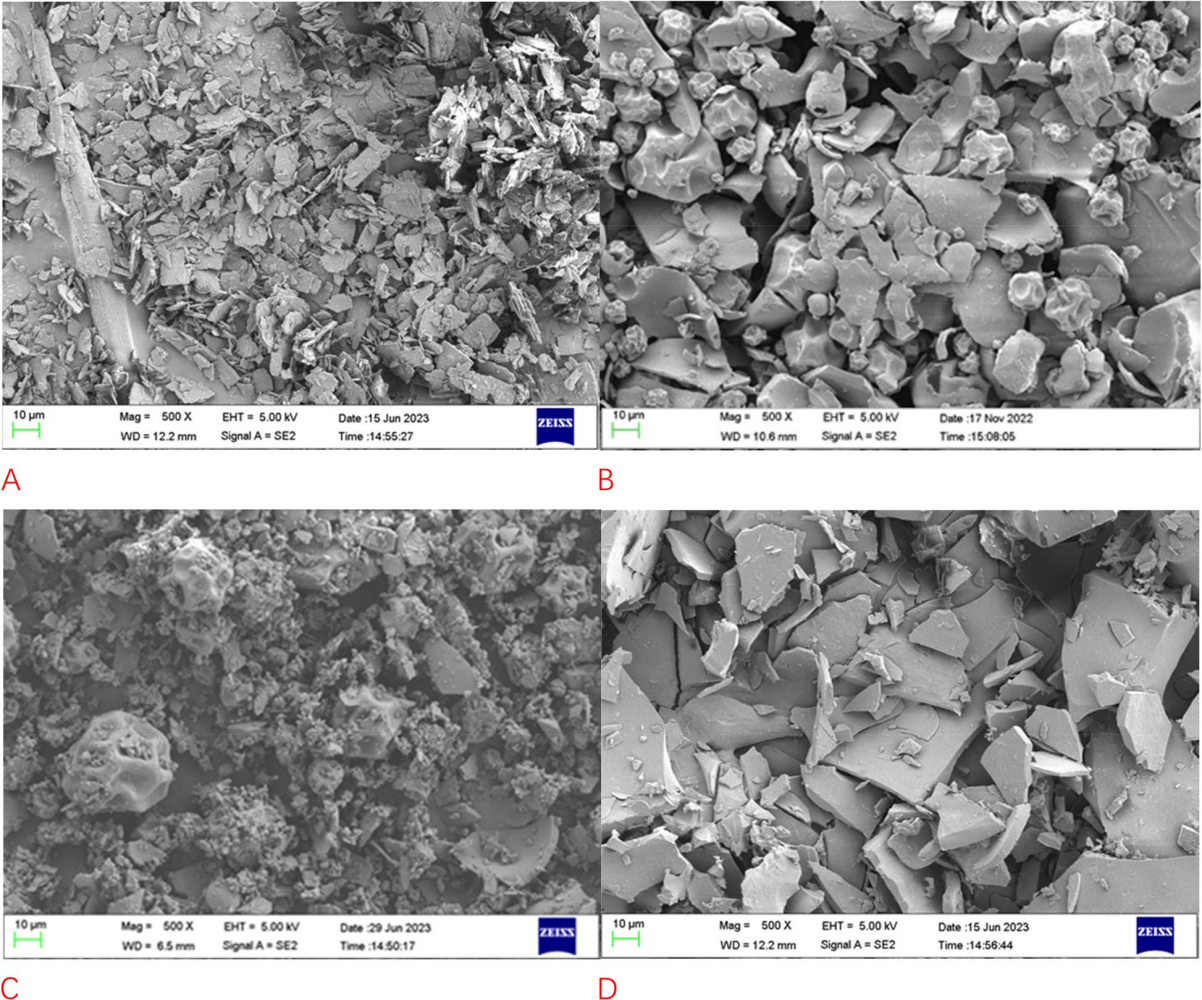
Scanning electron microscopic images of methyl-*β*-cyclodextrin, fenbendazole, their physical mixture and inclusion mixture

Scanning electron microphotograph was used to confirm the formation of the inclusion complex of fenbendazole and methyl-*β*-cyclodextrin. As shown in Fig. 3, fenbendazole is the fine flaky crystals (A), methyl-*β*-cyclodextrin is a ball-like crystal (B), in the morphology of the physical mixture, the fenbendazole and methyl-*β*-cyclodextrin crystals were observed respectively as the simple superposition of fenbendazole and methyl-*β*-cyclodextrin (C), while the particle size and morphology of the inclusion complex (D) were significantly different from the crystals of fenbendazole and methyl-*β*-cyclodextrin, indicated the new phase was formed.

**Fig. 4:**
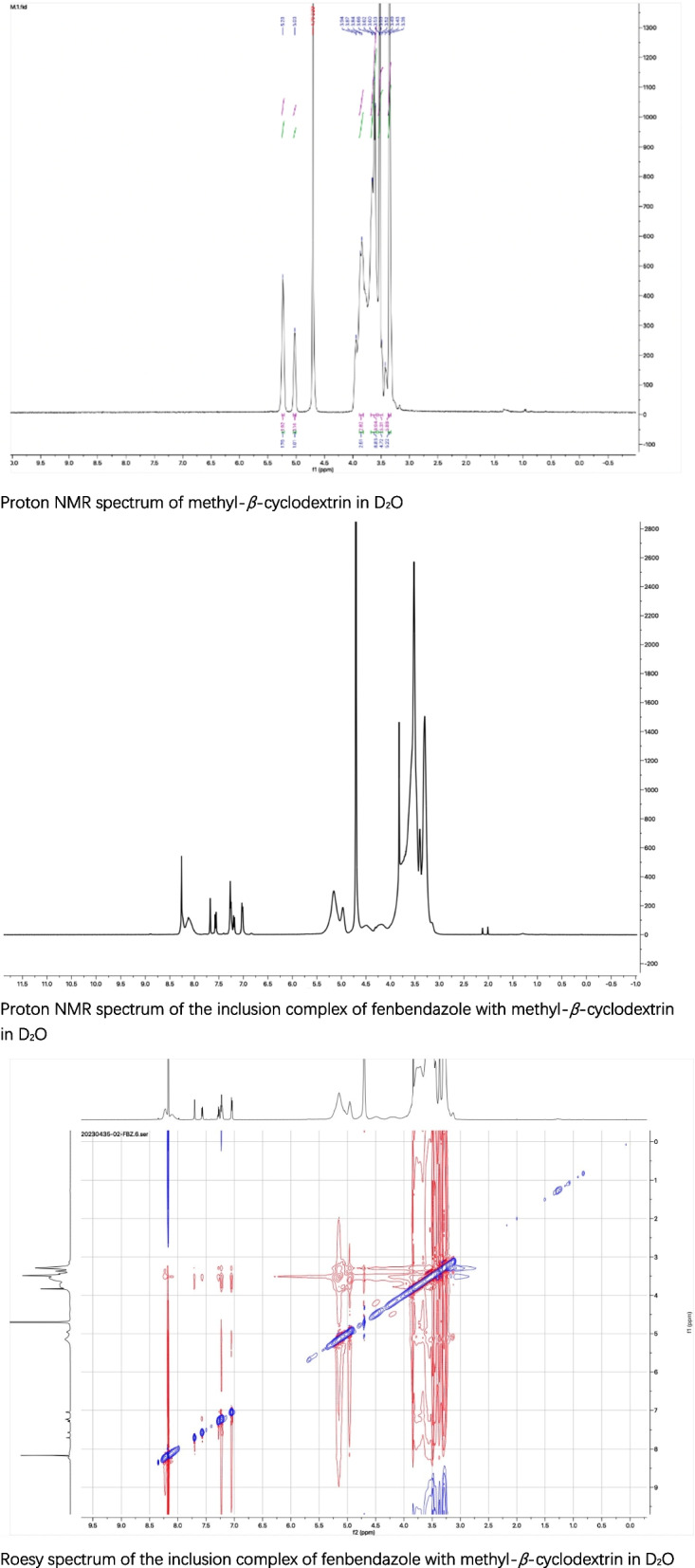
The proton NMR spectra of methyl-*β*-cyclodextrin and the inclusion complex in D_2_O, the Roesy spectrum of the inclusion complex in D_2_O

The changes in the morphology of the solid particles observed in DSC and SEM confirmed the complex formation. Nuclear magnetic resonance as the most effective method was used to study the space conformation of inclusion complex. The proton NMR spectra of methyl-*β*-cyclodextrin and its complex with fenbendazole in D_2_O, the Roesy spectrum of the inclusion complex in D_2_O were recorded (Fig. 4).

Proton NMR spectrum of methyl-*β*-cyclodextrin in D_2_O

Proton NMR spectrum of the inclusion complex of fenbendazole with methyl-*β*-cyclodextrin in D_2_O

Roesy spectrum of the inclusion complex of fenbendazole with methyl-*β*-cyclodextrin in D_2_O

**Fig. 5:**
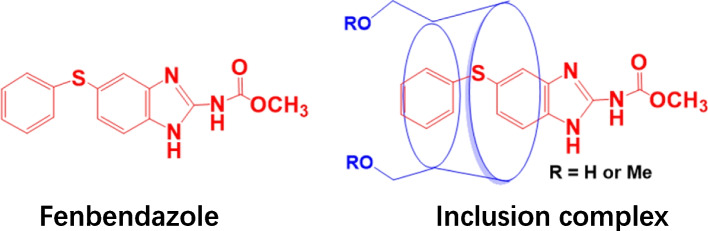
The structures of fenbendazole and its inclusion complex

Peaks in the proton NMR spectrum of the inclusion complex from fenbendazole in D_2_O were assigned as: 7.70 (1H, s), 7.55 (1H, d), 7.25 (3H, m), 7.23 (2H, d), 7.00 (2H, d), 3.80 (3H, s); the Roesy spectrum of the complex in D_2_O showed the interaction between the protons at 7.70 ppm, 7.55 ppm and 7.23 ppm from the phenyl group, at 7.25 ppm and 7.00 ppm from the benzimidazole motif in fenbendazole, and the protons from 3.25 ppm to 3.85 ppm in methyl-β-cyclodextrin, which confirm the formation of the inclusion complex. The ratio of the protons in fenbendazole and the protons at C-1 in methyl-*β*-cyclodextrin suggested the inclusion complex contained one molecular of methyl-*β*-cyclodextrin and one molecular of fenbendazole (Fig. 5).

**Fig. 6:**
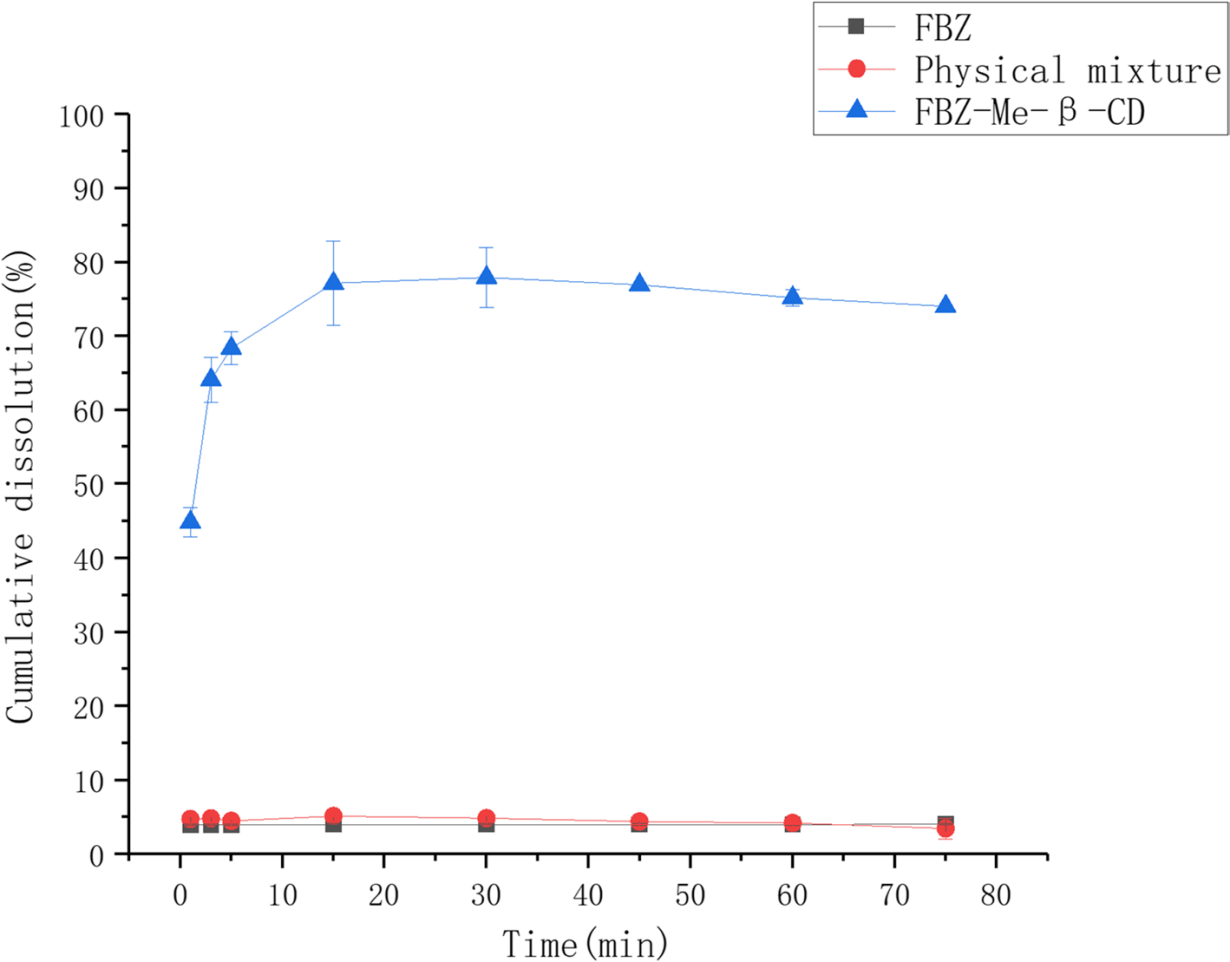
The dissolution rates of fenbendazole, the physical mixture of fenbendazole and methyl-*β*-cyclodextrin, and their inclusion complex

The dissolution rates of fenbendazole, the physical mixture of fenbendazole and methyl-*β*-cyclodextrin, and their complex were measured and shown in Fig. 6. Each experiment was repeated three times, and the average value of three experimental results was used to get the dissolution rate curves. The fenbendazole in complex can be 75% released in 15 min., which is 15 times better than that of fenbendazole alone or its physical mixture with methyl-*β*-cyclodextrin.

The *in vivo* pharmacokinetic study of fenbendazole and its inclusion complex in dogs was conducted. The dogs (12 dogs) were orally administrated with fenbendazole or its inclusion complex at a dose of 5 mg/kg, and blood samples were collected for up to 48 hours after administration.

Fenbendazole is metabolized in the liver to oxfendazole, which is anthelmintic agent and partially reduced back to fenbendazole in the liver and rumen. Fenbendazole sulfone is a minor metabolite of fenbendazole in plasma as a benzimidazole anthelmintic agent.

The HPLC conditions for analyzing the blank dog plasma, fenbendazole, oxfendazole and fenbendazole sulfone in which dog plasma does not interfere with the detection of fenbendazole, oxfendazole and fenbendazole sulfone was established. In the range of 0.025 μg/mL to 5 μg/mL, the peak areas in HPLC and the concentrations of fenbendazole, oxfendazole and fenbendazole sulfone in plasma solutions have a linear relationship, the standard curves are obtained, and regression equations of fenbendazole, oxfendazole and fenbendazole sulfone in plasma are summarized in Table 1.

**Table 1:** Regression equations for fenbendazole, oxfendazole and fenbendazole sulfone

Compounds	Regression equations
Fenbendazole	Y = 12.673 X + 80.417 (R^2^ = 0.9954)
Oxfendazole	Y = 2768.6 X + 25.592 (R^2^ = 0.996)
Fenbendazole sulfone	Y = 19.454 X + 92.153 (R^2^ = 0.9941)

Y: peak areas in HPLC, X: concentration, LLOD (S/N≥3): 0.05 µg/mL, LLOQ: 0.15 µg/mL

The HPLC samples including plasma samples were performed in the same HPLC instrument with the same C-18 column during a short period of time to avoid systematic errors, before and after the HPLC analysis, the standard solutions of fenbendazole, oxfendazole, and fenbendazole sulfone were checked by HPLC to confirm the stability of HPLC analysis conditions, and we did not use the internal standards for calibration.

**Fig. 7:**
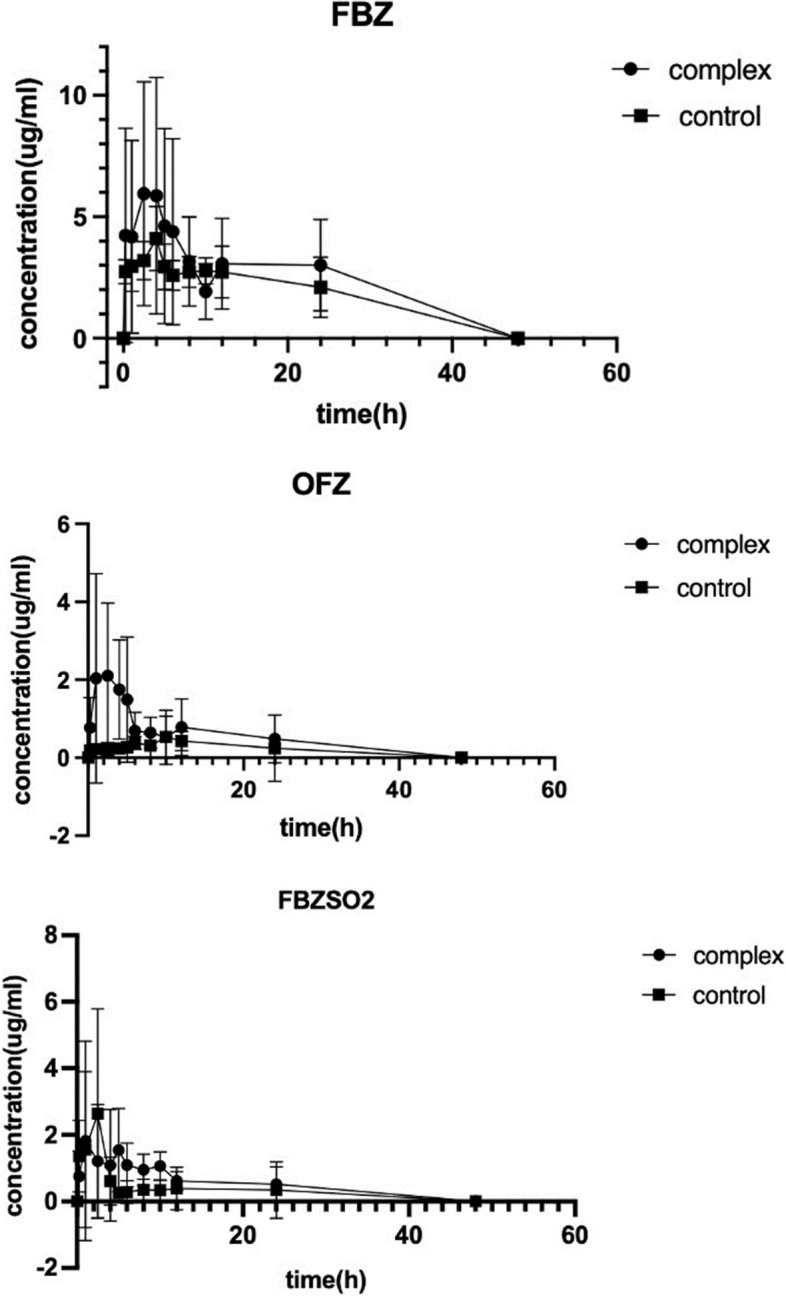
Curves of concentration-time of fenbendazole, oxfendazole, and fenbendazole sulfone in plasma from dogs dosed with fenbendazole or its methyl-*β*-cyclodextrin complex

The concentrations of fenbendazole, oxfendazole, and fenbendazole sulfone in plasma were determined by use of high-pressure liquid chromatography based on the standard cures and regression equations. The concentration-time curves in plasma were summarized in Fig. 7, the plasma pharmacokinetics profiles (mean ± SD) were determined by use of noncompartmental model and Phoenix WinNonlin 8 software and shown in Table 2.

**Table 2:** Pharmacokinetic parameters of fenbendazole, oxfendazole, and fenbendazole sulfone (mean ± SD) in fenbendazole group and complex group

		**Fenbendazole**		
**Parameters**	Units	FBZ	OFZ	FBZSO_2_
*T*_***max***_ 2.45±2.02		h	8.17±9.11	19.67±6.16
*C*_*max*_	μg	4.41±1.27	1.38±0.69	4.11±3.03
*T*_*1/2*_ 2.43±2.05		h	33.19±8.47	10.25±5.46
AUC_0~48_ 11.93±8.68		h*μg/mL	60.68±19.91	13.72±6.62
MRT_0~48_ 4.26±1.73		h	19.51±10.43	15.75±4.59
		**Fenbendazole Complex**		
**Parameters**	Units	FBZ	OFZ	FBZSO_2_
*T*_**max**_ 4.92±2.83		h	4.50±1.96	3.58±2.46
*C*_*max*_	μg	7.68±4.75	4.13±1.97	3.65±2.55
*T*_*1/2*_ 10.07±7.21		h	13.64±1.51	15.25±11.88
AUC_0~48_ 20.24±13.97		h*μg/mL	83.65±51.63	20.54±8.82
MRT_0~48_ 16.68±10.05		h	15.71±4.61	25.19±17.27

Through complexation with methyl-*β*-cyclodextrin, the *C*_max_ and *T*_max_ of fenbendazole were increased from 4.41±1.27 µg at 8.17 h to 7.68±4.75 µg, AUC_0~48_ was increased from 60.68±19.91 h*µg/mL to 83.65±51.63 h*µg/mL, and the relative bioavailability is 138%; the *C*_max_ and *T*_max_ of the major metabolic anthelmintic agent oxfendazole was increased from 1.38±0.69 µg at 19.67±6.16 h to 4.13±1.97 µg at 3.58±2.46 h, AUC_0~48_ was increased from 13.72±6.62 h*µg/mL to 20.54±8.82 h*µg/mL, the relative bioavailability is 149%, the half-life time *T*_1/2_ was prolonged from 10.25±5.46 h to 15.25±11.88 h; the *C*_max_ and *T*_max_ of the minor metabolic anthelmintic agent fenbendazole sulfone was decreased from 4.11±3.03 µg at 2.45±2.02 h to 3.65±2.55 µg at 4.92±2.83 h, however, the AUC_0~48_ was increased from 11.93±8.68 h*µg/mL to 20.24±13.97 h*µg/mL, the relative bioavailability is 169%, and half-life time *T*_1/2_ was prolonged from 2.43±2.05 h to 10.07±7.21 h

Overall, enlarged AUC_0~48_ of fenbendazole, oxfendazole and fenbendazole sulfone, enlarged *C*_max_ of fenbendazole and oxfendazole, prolonged* T*_1/2_ of oxfendazole and fenbendazole sulfone will allow less drug dose for the same therapeutic effect by using the inclusion complex, more important, as fenbendazole is showing anti-tumor activity, it is expected to promote further investigations toward repurposing of this potent compound as cancer drug.

## Conclusion

Through single factor and orthogonal strategy, the complexation conditions for preparing the inclusion complex of fenbendazole with methyl-*β*-cyclodextrin were found. The prepared inclusion complex with 29.2 % of inclusion rate and 89.5% of inclusion yield could increase the water solubility of fenbendazole from 0.3 µg/mL to 20.21 mg/mL. It’s *in vitro* and *in vivo* pharmacokinetic study results indicated less drug is needed to reach the same therapeutic effect by using this complex. More important, as an anti–cancer agent, this complex meets the cancer drug’s water solubility requirements (5–10 mg/mL), can be used as potential anti-cancer drug.

## Experimental part

### Materials and instruments

Fenbendazole with 99.9% purity, *β*-cyclodextrin and 2-hydroxypropyl-β-cyclodextrin (average Mw: 1460) were purchased Fenbendazole (content 98%) was purchased from Shanghai Aladdin Biochemical Technology Co., Ltd. (Shanghai, China), γ-cyclodextrin, 2-hydroxypropyl-γ-cyclodextrin (DS: 4–6), and methyl-*β*-cyclodextrin (DS: 11–13) were purchased from Shanghai Macklin Biochemical Co., Ltd. (Shanghai, China).

Dogs were obtained from Guangdong Medical Laboratory Animal Center, the blank dog plasma was received from Guangzhou Rui-Te Co. Ltd. (Guangzhou, Guangdong, China) and the low-fat dog foods were obtained from Shanghai Jibai Chong Industrial Co., Ltd. (Shanghai, China).

### Determination of the maximum UV wavelength of fenbendazole

The solution of fenbendazole in ethanol (0.1 mg/mL) and solutions of *β*-cyclodextrin, HP-*β*-cyclodextrin, methyl-*β*-cyclodextrin, *γ*-cyclodextrin or HP-*γ*-cyclodextrin in water with concentrations of 0.1 mg/mL were scanned by an ultraviolet-visible spectrophotometer (J51903001 from Shanghai Jinghua Technology Instrument Co., Ltd., Shanghai, China) in the wavelength range of 200 nm-400 nm by using the distilled water as a blank, It was found that fenbendazole had the maximum absorption at 296 nm, while cyclodextrins had no absorption, 296 nm was chosen as the determining wavelength of fenbendazole in HPLC analysis.

### HPLC analysis conditions

Fenbendazole was analyzed by Agilent 1260 infinity high performance liquid chromatography instrument ((LC-15C high performance liquid chromatograph from Shimadzu Enterprise Manage-ment China Co., Ltd., Shenzhen, China) on C18-Ecosil chromatographic column 250 mm × 4.6 mm (5 μm, Guangzhou Lubex Scientific Instrument Co., Ltd., Guangzhou, P. R. China) by using acetonitrile-water (70%:30%) as mobile phase at 25°C with UV detector at 296 nm, the flow rate was 1 mL/min., and the injection volume was 10 μL.

### The preparation of HPLC samples

The sample was dissolved in certain solvent including water or plasma, or other solvent system, Shaked for few minutes and filtered through the 0.22 μm membrane for HPLC analysis.

### Establishment of HPLC standard curve of fenbendazole

The solutions of fenbendazole in ethanol with concentrations of 0.01, 0.02, 0.04, 0.06, 0.08 and 0.1 mg/mL were analyzed by HPLC with UV detector at 296 nm, based the peak areas and the concentrations, the standard curve is established, and regression equation were obtained as follow:

### Phase solubility study of fenbendazole

Excess fenbendazole was added to the solutions of *β*-cyclodextrin, methyl-*β*-cyclodextrin, HP-*β*-cyclodextrin, *γ*-cyclodextrin and HP-*γ-*cyclodextrin with concentrations of 5, 10, 15, 20, 25, and 30 mmol/mL respectively, stirred at room temperature for 48 hours, filtrated and analyzed by HPLC, based on the content of fenbendazole in solutions, the phase solubility results were obtained. Each reaction was repeat three times.

### Preparation of the inclusion complex of fenbendazole with methyl-β-cyclodextrin

Fenbendazole was dissolved in pure formic acid and added to the solution of *methyl-β-cyclodextrin* in water with stirring with certain ratio of drug and cyclodextrin, stirring speed, reaction temperature and reaction time. The solution was kept in a refrigerator at 4 °C for several hours, the solvents were removed by rotary evaporation, the residue was dissolved in water, filtered, and freeze-dried under reduced pressure to obtain the inclusion compound as powder.

### Preparation of the physical mixture of fenbendazole and methyl-β-cyclodextrin

The appropriate amounts of fenbendazole and methyl-*β*-cyclodextrin (1:1 ratio) were grind and mixed to get the physical mixture of fenbendazole and methyl-*β*-cyclodextrin.

### Content determination of fenbendazole in complex

Excess fenbendazole/methyl-*β*-cyclodextrin inclusion complex was dissolved in ultrapure water (10 mL), after ultrasonicate and shake for 2 hours at 37°C, the saturated aqueous solution was centrifuged, filtered, and analyzed by HPLC, based on the fenbendazole HPLC standard curve and regress equation, the content of fenbendazole in inclusion complex can be obtained.

### Determination of inclusion rate and inclusion yield

The inclusion rate and yield of inclusion compound were used to evaluate the inclusion effects of complexations. The inclusion rate and yield of fenbendazole inclusion compound could be calculated by the following formulas:

Inclusion yield (%) = (fenbendazole complex)/[(fenbendazole)+(methyl-*β*-CD)]x 100%

Inclusion ratio (%) = [(fenbendazole in complex) / (fenbendazole)] x 100%

### Differential scanning calorimetry study

Fenbendazole inclusion complex, fenbendazole and methyl-*β*-cyclodextrin, and their physical mixture around 5 to 10 mg were used for Differential scanning calorimetry (the differential scanning calorimeter DSC3 from Mettler Toledo, Columbus, OH, USA) analysis under a N_2_ atmosphere with an air flow rate of 20 mL/min., an empty aluminum crucible was used as the reference cell and another empty aluminum crucible was used as the sample cell. The scanning conditions were set to a scanning speed was set up as 10 °C/min.

### Scanning electron microscopic image study

Scanning electron microscope (Merlin from German Zeiss Company, Oberkochen, Germany) was used to observe the fenbendazole, methyl-*β*-cyclodextrin, their inclusion complex, and physical mixture with the voltage of 5kV, samples were mounted on copper plates with conductive tape and gold blasted.

### NMR study of fenbendazole inclusion complex

The proton NMR spectra of fenbendazole complex in D_2_O, methyl-*β*-cyclodextrin in D_2_O Bruker 400 spectrometer (Bruker, Karlsruhe, Germany), the Roesy spectrum of fenbendazole complex in D_2_O in Bruker 600 spectrometer (Bruker, Karlsruhe, Germany) were recorded.

### Dissolution rate determination

The *in vitro* dissolution rate was measured on the dissolution tester RC-6 (from Tianjin Xintianguang Analytical Instrument Tech-nology Co., Ltd., Tianjin, China) according to the method specified in Appendix 160 of the first part of the 2015 edition of the Chinese Veterinary Pharmacopoeia. Fenbendazole (100 mg), the inclusion complex of fenbendazole and methyl-*β*-cyclodextrin (containing 100 mg of drug), the physical mixture of fenbendazole and methyl-*β*-cyclodextrin (containing 100 mg of the drug) in a reaction vessel was added 900 mL of ultrapure water respectively, ultrasonic degassed for 15 min., stirred with the speed of 100±3 r/min. at 37±0.3°C. At 2, 5, 10, 15, 20, 30, 45 and 60 minutes, sample (3 mL) was collected and filtered through a 0.22 μm micropore syringe filter, at the same time, 3 mL of isothermal ultra-dissolution medium (water) was added to the reaction vessel. Samples were analyzed by HPLC, based on the peak area data in HPLC, fenbendazole HPLC standard curve ang regression equation, the cumulative dissolution rate was obtained. Each test was repeated three times.

### Establishment of the stand curves of fenbendazole, oxfendazole, and fenbendazole sulfone in plasma

The solutions of fenbendazole, oxfendazole, and fenbendazole sulfone in acetonitrile (0.5 mL) with concentrations of 10 μg/mL, 5 μg/mL, 1 μg/mL, 0.2 μg/mL, 0.1 μg/mL and 0.05 μg/mL were added to blank dog plasma (0.5 mL) in a plastic centrifuge tube respectively. After vortexing and vibration, the mixtures were extracted with dichloromethane and ethyl acetate, blown with nitrogen, dissolved in methanol (1 mL), and analyzed by HPLC at 296 nm on a Shim-packVP-ODS C18 chromatographic column (250L×4.6mm), to give the standard curves and regression equations of fenbendazole, oxfendazole, and fenbendazole sulfone in plasma as follow:

### In vivo Pharmacokinetic studies

Twelve healthy adult dogs around 5±0.1kg with half male and half female were randomly divided into two groups named as the fenbendazole group and inclusion group (six for each group), weighed, numbered, fed for one week in a new warm, clean, and ventilated environment, fasted for one day before drug administration, orally dose the fenbendazole or its inclusion complex with water at a dose of 5 mg/kg, and the blood sample (2 mL) was collected through forearm vein at 0.00, 0.25, 0.5, 0.75, 1, 2, 4, 6, 10, 12, 24 and 48 hours after administration, transferred Immediately to a heparinized test tube, centrifuged at 3500 rpm for 10 min., stored in a -20°C in a refrigerator. During the sampling period, the dogs were fed with low-fat dog food and normal drink water.

The plasma sample was then thawed to room temperature, extracted with CH_2_Cl_2_ (1 mL), shaken for 4 min., and centrifuged at 13000 r/min. for 10 min. two times. The combined dichloromethane solution in a 10 mL sterilized centrifugal tube was evaporated by blowing the nitrogen gas at room temperature, added 0.5 mL of methanol, shaken for 4 min., centrifuged for 10 min. at 13000 r/min speed, filtered through 0.22μm filter, and analyzed by HPLC.

### Statistical analysis

Each experiment was repeated three times. The test results are expressed in the form of "mean ± standard deviation (MEAN ± SD)". Graphpad Prism 9 software was used to draw graphs and perform statistical analysis on the data. *P*≤0.05 means the difference is considered statistically significant.

## Data Availability

No datasets were generated or analysed during the current study.

## References

[1] Accioni F, Caballero-Casero N, García-Gómez D, Rubio S (2019). Restricted access volatile supramolecular solvents for single step extraction /cleanup of benzimidazole anthelmintic drugs in milk prior to LC-MS /MS. J. Agric. Food Chem..

[2] Mrkvová Z, Uldrijan S, Pombinho A, Bartůněk P, Slaninová I (2019). Benzimidazoles downregulate Mdm2 and MdmX and activate p53 in MdmX overexpressing tumor cells. Molecules..

[3] Duan Q, Liu Y, Rockwell S (2013). Fenbendazole as a potential anticancer drug. Anticancer Res..

[4] Dogra N, Kumar A, Mukhopadhyay T (2018). Fenbendazole acts as a moderate microtubule destabilizing agent and causes cancer cell death by modulating multiple cellular pathways. Sci. Rep..

[5] Tang Y, Liang J, Wu A, Chen Y, Zhao P, Lin T (2017). Co-delivery of trichosanthin and albendazole by nano-self-assembly for overcoming tumor multidrug-resistance and metastasis. ACS Appl. Mater. Interfaces..

[6] Chu SW, Badar S, Morris DL, Pourgholami MH (2009). Potent inhibition of tubulin polymerisation and proliferation of paclitaxel-resistant 1A9PTX22 human ovarian cancer cells by albendazole. Anticancer Res..

[7] Gu J.l, Zhao H, Liu L, Yang G (2020). Preparation and characterization of the fenbendazol-β-cyclodextrin inclusion. Chem Rese  Appli.

[8] Jang-Ha R, James D, Sandra EK, James CR, Leonard IW (2013). A water-soluble fenbendazole formulation for treating pinworm infections in laboratory animals. Drug Delivery Letters.

[9] Effectiveness of anthelmintics: general recommendations VICH GL7. FDA; Rockville, USA: 2001. Guidance for industry. Available via http://fda.gov/downloads/GFI-Anthelmintics-general-recommendations. Fenbendazole (WHO Food Additives Series 29). Available via http://www.inchem.org/documents/jecfa/jecmono/v29je04.htm.

[10] Rodrigues LNC, Tavares ACM, Ferreira BT, Reis AKCA, Katiki LM (2019). Inclusion complexes and self-assembled cyclodextrin aggregates for increasing the solubility of benzimidazoles. Braz. J. Pharm. Sci..

[11] Artem O, Surov NA, Vasilev MV, Vener OD, Parashchuk AV, Churakov OV, Magdysyuk GLP (2021). Pharmaceutical salts of fenbendazole with organic counterions: structural analysis and solubility performance. Crystal Growth & Design..

[12] Ivan AA, Salavat SK, Konstantin MS, Alexander VD, Elizaveta SM, Anastasiya IV, Irina MO, Nataliya VD (2019). Influence of mechanochemical technology on anthelmintic efficacy of the supramolecular complex of fenbendazole with polyvinylpyrrolidone. J. Adv. Vet. Anim. Res..

[13] María EM, Manuel I, Laura C, Alejandro JP, Beatriz M, Ricardo F, Santiago P, Luis IA, Laura D (2022). Improving the in vitro dissolution rate and pharmacokinetic performance of fenbendazole in sheep using drug nanocrystals. Research in Veterinary Science.

[14] Esfahani MKM, Alavi SE, Cabot PJ, Islam N, Izake EL (2021). PEGylated mesoporous silica nanoparticles (MCM-41): a promising carrier for the targeteddelivery of fenbendazole into prostate cancer cells. Pharmaceutics..

[15] Shin YB, Choi JY, Shin DH, Lee JW (2023). Anticancer evaluation of methoxy poly(Ethylene Glycol)-b-Poly(Caprolactone) polymeric micelles encapsulating fenbendazole and rapamycin in ovarian cancer. Int J Nanomed.

[16] Hickey T, Kreutzer D, Burgess DJ, Moussy F (2002). Dexamethasone/PLGA microspheres for continuous delivery of an anti-inflammatory drug for implantable medical devices. Biomaterials.

[17] Chang C. S, Ryu  J. Y, Choi J. K, Cho Y. J, Choi J. J, Hwang J. R, Choi J. Y, Noh J. J, Lee C M, Won J E, Han H. D, Lee J. W (2023). Anti-cancer effect of fenbendazole-incorporated PLGA nanoparticles in ovarian cancer. J Gynecol Oncol.

[18] Bezerra G. S. N., de Lima T. A. de M., Colbert D. M., Geever J., Geever L., Formulation and evaluation of fenbendazole extended-release extrudes processed by hot-melt extrusion. Polymers. 2022;14(19): 4188. 10.3390/polym14194188. 10.3390/polym14194188 PMC9573241 36236135

[19] Bezerr G. S. N., Moritz V. F., de Lima T. A. de M., Colbert D. M., Geever J., Geever L., Compatibility study between fenbendazole and polymeric excipients used in pharmaceutical dosage forms using thermal and non-thermal analytical techniques. Analytica. 2022;3(4): 448-461. 10.3390/analytica3040031.

[20] Bezerra GSN, De Lima GG, Colbert DM, Halligan E, Geever J, Geever L (2023). Micro-injection moulding of PEO/PCL blend–based matrices for extended oral delivery of fenbendazole. Pharmaceutics.

[21] Ding Y, Yu B, Zhang J, Ding C, Zhang Z, Xu S, Li L, Yu H (2022). Tilmicosin/γ-cyclodextrin complexation through supercritical carbon dioxide assistance and its pharmacokinetic and antibacterial study. Eur. J. Pharm. Biopharma..

[22] Ding Y, Yu B, Zhou S, Ding C, Zhang Z, Xu S, Xu Z (2023). Improvement of solubility and pharmacokinetic profile of hepatoprotector icariin through complexation with HP-γ-cyclodextrin. Front. Pharmacol..

[23] Ding Y, Zhang Z, Ding C (2023). Shufeng Xu Zhe Xu, The Use of Cyclodextrin inclusion complexes to increase the solubility and pharmacokinetic profile of albendazole. Molecules..

[24] Ding Y, Cui W, Zhang Z, Ma Y, Ding C, Lin Y, Xu Z (2023). Solubility and pharmacokinetic profile improvement of griseofulvin through supercritical carbon dioxide-assisted complexation with HP-γ-cyclodextrin. Molecules.

